# Glycemic dysregulation and cognitive impairment in aging adults: a cross-sectional study with amyloid biomarker correlation

**DOI:** 10.3389/fragi.2026.1696711

**Published:** 2026-04-28

**Authors:** R. Rajalakshmi, C. M. Ramya, Rimshia Naaz, SubbaRao V. Madhunapantula, Chinnappa A. Uthaiah, Paramahans V. Salimath

**Affiliations:** 1 Department of Physiology, JSS Medical College, JSS Academy of Higher Education & Research (JSS AHER), Mysore, Karnataka, India; 2 Center of Excellence in Molecular Biology and Regenerative Medicine (CEMR) Laboratory (DST-FIST supported center and ICMR Collaborating Center of Excellence – ICMR-CCoE), Department of Biochemistry, JSS Medical College, JSS Academy of Higher Education & Research (JSS AHER), Mysore, Karnataka, India; 3 Special Interest Group in Cancer Biology and Cancer Stem Cells (SIG-CBCSC), JSS Academy of Higher Education & Research, Mysore, Karnataka, India; 4 Superannuated from JSS Academy of Higher Education & Research, Mysore, Karnataka, India

**Keywords:** aging, amyloid-βeta, cognition impairment, dyslipidemia, insulin resistance, neurodegeneration, type 2 diabetes mellitus

## Abstract

**Background:**

Type 2 Diabetes Mellitus (T2DM) is a rising health concern, particularly affecting the elderly population. Beyond its well-established metabolic consequences, growing evidence suggests a strong association between type 2 diabetes mellitus and cognitive decline. The core features of diabetes, viz., chronic hyperglycemia and insulin resistance (IR), not only contribute to the neurodegenerative changes in the brain but also promote the generation and accumulation of amyloid-β (a hallmark feature in Alzheimer’s disease). These molecular changes triggered by T2DM play a pivotal role in the onset of cognitive damage. In this study, we have explored the interplay between glycemic status, cognitive performance, and plasma amyloid-β (Aβ) levels in an ageing population.

**Methodology:**

A cross-sectional study was conducted among 396 individuals aged 51–80 years. Based on their HbA1c levels, the study participants were categorized into four glycemic groups: individuals without diabetes, individuals with prediabetes, individuals with diabetes, and individuals with uncontrolled diabetes. Cognitive function was evaluated using the Modified Mini-Mental State Examination (3MSE). Fasting glucose, insulin, Homeostatic Model Assessment (HOMA)-IR, lipid profiles, and plasma Aβ 1–40 and Aβ 1–42 were measured. Statistical analyses were carried out using Chi-square tests, logistic regression, Spearman correlation, and Receiver Operating Characteristic (ROC) curve analysis.

**Results:**

A comparative assessment in the study revealed the prevalence of cognitive impairment across all glycemic groups, with 40.15% of participants overall affected. It was observed that the prevalence increased with the glycemic index, with 26.97% of individuals without diabetes affected, 35.92% of individuals with prediabetes, 55.03% of individuals with diabetes under control, and 70.91% of individuals with uncontrolled diabetes (p < 0.001). Logistic regression indicated progressively higher odds of cognitive impairment with worsening glycemic control (OR for individuals with uncontrolled diabetes ≈6.87, p < 0.001). Age and HbA1c were significantly inversely correlated with 3MS scores. Plasma Aβ 1–40 and Aβ 1–42 levels were elevated in individuals with diabetes groups, while the Aβ 1–42/Aβ 1–40 ratio was positively associated with cognitive performance. The ROC curve analysis of the logistic regression indicated an Area Under Curve (AUC) of 0.76, suggesting good predictive capability.

**Conclusion:**

Impaired glycemic status was found to be strongly associated with increased cognitive decline, alongside altered amyloid biomarker profiles, in elderly populations. These findings suggest the heightened importance of metabolic homeostasis and also underscore the critical need for early metabolic interventions and cognitive screening in the individuals with diabetes to mitigate the risk of early neurodegeneration.

## 1 Introduction

Ageing is an irreversible physiological phenomenon that brings about its own bodily and cognitive changes, which in turn depend on lifestyle and environmental factors (Salvatore, 2020). The elderly population cohort is increasing steadily worldwide due to various clinical advancements, resulting in declining mortality rates and improved life expectancy (Mathers et al., 2015). According to a 2011 census conducted by the Indian National Population Committee, the elderly population numbered approximately 104 million, accounting for 8.6% of the total population. Notably, the older women’s population (53 million) outnumbers the older men’s (51 million) (Hashmi, 2018). According to the India Ageing Report 2023, produced by the United Nations Population Fund (UNFPA) in partnership with the International Institute for Population Sciences (IIPS), it is projected that India’s elderly population will reach 173 million by 2026 and surpass 300 million by 2050 (Debnath et al., 2024). This vulnerable section of the elderly population is facing health system concerns from non-communicable diseases, including diabetes mellitus, cardiovascular disorders, cancer, respiratory ailments, and mental health issues (Sharma et al., 2024).

Type 2 Diabetes Mellitus is a chronic metabolic condition characterized by persistent hyperglycemia, insulin resistance, and hyperinsulinemia (Galicia-Garcia et al., 2020). According to the World Health Organization (WHO), diabetes represents one of the significant global health concerns. India alone accounts for approximately 77 million individuals with type 2 diabetes mellitus, along with an estimated 25 million people in the prediabetic stage (Pradeepa and Mohan, 2021). Diabetes mellitus, if left uncontrolled, can further lead to complications associated with increased morbidity and mortality rates. T2DM burdens families and society economically, as it requires access to substantial healthcare on time (Cannon et al., 2018). Glycemic status indicates chronic glycemic control, classified by glycated hemoglobin (HbA1c) thresholds as defined by the American Diabetes Association: nondiabetic <5.7, prediabetic 5.7–6.4, diabetic 6.5–7.9, and uncontrolled diabetic ≥8.0. This stratification reflects a continuum of metabolic disturbance rather than a diabetic diagnosis (Qaseem et al., 2018).

T2DM can interrupt normal glucose metabolism, insulin signaling pathways, and mitochondrial function, which can impact neuronal health and increase the risk of cognitive deterioration and Alzheimer’s disease. Several clinical and epidemiological studies have evidenced a strong association between T2DM and increased likelihood of cognitive impairment and dementia, among the elderly in particular (Cholerton et al., 2016; Bello-Chavolla et al., 2019). Cognition, a psychological domain, encompasses a spectrum of cognitive functions, including attention capacity, learning mechanisms, memory retention, language skills, visuospatial abilities, and executive functions such as decision-making, planning, and judgment (Harvey, 2019). Cognitive disorders, including dementia, amnesia, and executive motor skill disturbances, primarily cripples the cognitive function domains. With improved life expectancy, the prevalence of cognitive disorders is rising rapidly both in India and globally. According to the Dementia in India Report 2020, published by the Alzheimer’s and Related Disorders Society of India (ARDSI), approximately 5.3 million Indians aged over 60 years (roughly one in 27 individuals) suffer from dementia. Projections suggest this number will rise dramatically by 2030 (Lee et al., 2023).

Alzheimer’s disease (AD), a common form of dementia, is a late-onset neurodegenerative disorder featured by the accumulation of hallmark pathological biomarkers, amyloid beta (Aβ) peptides, and neurofibrillary tangles (NFTs) (Wang et al., 2023). Evidence from recent studies has implicated dysregulated glucose metabolism, insulin resistance, mitochondrial cell dysfunction, and metabolic byproducts of oxidative stress in the pathogenesis of AD. Specifically, disruptions in insulin signaling pathways have been associated with decreased cognitive performance in individuals affected with AD (Yoon et al., 2023; Berlanga-Acosta et al., 2020). Serum levels of amyloid beta (Aβ), particularly the Aβ42 isoform, reflect the balance between cerebral production, clearance, and degradation of Aβ. Even the Aβ42/Aβ40 ratio resonates as a potential biomarker of AD, with a higher ratio value indicating greater Aβ42 accumulation in the brain (Schindler et al., 2019; Janelidze et al., 2016). There are also a few studies that have demonstrated significantly lower levels of both Aβ42 and Aβ40 in the plasma of diabetic patients compared to those of controls, but have shown elevated Aβ42/Aβ40 ratio values in individuals with diabetes (Peng et al., 2020; Fandos et al., 2017). Among patients with both T2DM and cognitive impairment—such as mild cognitive impairment (MCI) or AD—altered serum levels of Aβ42 have been observed. Although brain Aβ accumulation is a hallmark of AD, the role of circulating Aβ levels in disease progression remains under investigation. Current evidence suggests that elevated serum Aβ42 levels may be associated with cognitive decline in individuals with type 2 diabetes mellitus (T2DM) (Risacher et al., 2019; Liu et al., 2021; Schraen-Maschke et al., 2024). Research linking diabetes and cognitive decline in the Indian elderly population is scarce, particularly regarding glycemic dysregulation and circulating amyloid isoforms. Previous studies have mainly focused on cognitive screening in diabetic cohorts without examining biochemical amyloid correlates, while global research often overlooks metabolic profiling in Alzheimer’s cohorts. This study addresses that gap by analyzing plasma Aβ_1-40_, Aβ_1-42_, and their ratio (Aβ_1-42_/Aβ_1-40_) across graded glycemic categories within a South Indian aging cohort.

Given this background, the current research proposes that there is a relationship between type 2 diabetes mellitus and cognitive decline. Therefore, the objective of this study is to explore the interrelationships between glycemic status, cognitive function, and amyloid beta levels in the elderly South Indian population.

## 2 Materials and methods

### 2.1 Study design and population

The cross-sectional observational study was conducted at JSS Hospital, a teaching-cum-training tertiary care hospital, and was approved by the Institutional Ethics Committee of JSS Medical College, Mysore (JSSMC/IEC/14/3712/2016- 17). To reduce the risk of bias associated with recruiting participants from hospitals, a multifaceted approach was employed, involving inpatient, outpatient, and community settings. A total of 450 individuals were screened for the study, which encompassed clinically stable visitors and bystanders in hospitals, all aged between 50 and 80 years, regardless of gender. However, the demographic trends observed in older patients showed more female attendees for outpatient clinics. Totally, 396 participants were enrolled based on predefined inclusion and exclusion criteria. The study participants with a history of more than 5 years of Type 2 DM and normal subjects without diabetes were included in the study. Those subjects with obesity, coronary heart disease, cardiac failure, cerebrovascular events, hypoglycaemia, other metabolic disorders, autoimmune diseases, neurological disorders, and carcinomas were excluded from the study. Normal individuals without a prior history of diabetes were tested for their glycemic index and considered a healthy control group. Out of 450 individuals screened, 54 dropped out due to any one or more of the following reasons:

24 subjects with diabetes-related complications, 10 with coronary artery disease, 5 with endocrine disorders, 2 with stroke, 10 with psychiatric illness, and 3 with malignancies. Before enrollment in the study, informed written consent (prepared in two languages: English and the local language, i.e., Kannada) was obtained from all participants. A trained physician conducted a comprehensive clinical history and physical examination. Anthropometric records, including measurements of body height in meters, weight in kilograms, and waist and hip circumference in centimeters, were recorded using a standard procedure.

### 2.2 Blood sample collection

A phlebotomist collected approximately 6.0 mL of venous blood in the fasting state in an EDTA-containing blood collection tube (BD Vacutainers, Cat no: 367,863) for the analysis of biochemical parameters. The samples collected were processed by separating the serum through centrifugation at 4,000 rpm for 15 min. The separated serum was aliquoted and stored in −80 °C to preserve sample integrity and for further use.

### 2.3 Measurement of fasting blood sugar, fasting insulin, hemoglobin A1c, and HOMA-IR

(a) The fasting blood sugar levels were measured using the Gluc3-Glucose hexokinase kit (Roche, Catalogue no: 04404483–190) following instructions provided by the manufacturer on a fully automated Cobas c 501 auto analyzer (Roche, USA). (b) Fasting serum insulin levels were measured using the Elecsys Insulin kit (Roche, Catalogue No. 12017547-122) on a fully automated electro immunoassay analyzer (Cobas e 601, Roche, USA). (c) The hemoglobin A1c were analyzed using a D-10 hemoglobin A1c kit (Roche, Catalogue no: 220-0101) on the Bio-Rad D-10 hemoglobin system (Bio-Rad, USA). (d) HOMA-IR: Insulin resistance was calculated using the Homeostatic Model Assessment (HOMA-IR) formula: HOMA-IR = Fasting Insulin (μIU/mL) × FBS (mg/dL)/405.

### 2.4 Measurement of lipid profile

The lipid profile markers were assessed using their respective kits. Triglycerides (Roche, Catalogue no: 20767107–322), HDL (Roche, Catalogue no: 04399803-190), LDL (Roche, Catalogue no: 07005717–190), Total Cholesterol (Roche, Catalogue no: 03039773–190) using a fully automated Cobas c 501 auto analyzer (Roche, USA).

### 2.5 Measurement of amyloid beta

The Aβ 1–40 and Aβ 1–42 was measured using a highly sensitive competitive ELISA kit (a) Aβ “(1–40)” (Cloudclone, Catalogue no: CEA864Hu) (b) Aβ “(1–42)” (Cloudclone, Catalogue no: HEA946Hu) following the manufacturer’s protocol. The ratio of Aβ 1-42/Aβ 1-40 indicated the risk of alzheimer’s disease. The lower the ratio, the higher the amyloid burden, resulting in an increased risk.

### 2.6 Cognitive assessment

**FIGURE 1:**
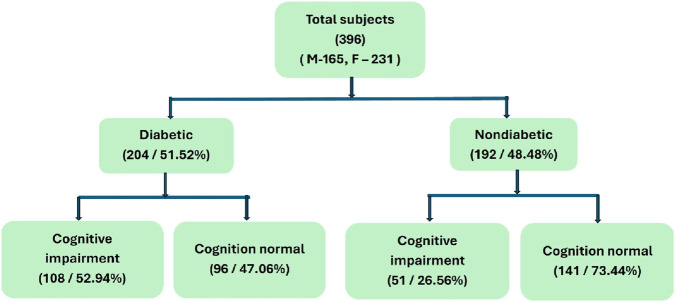
Schematic flow chart for recruitment and classification of study subjects based on diabetes condition and cognition status (3MSE).

Cognitive performance was assessed using the Modified Mini- Mental State Examination (3MSE), which is an improved version of MMSE (Mini-Mental State Exam). The 3MS is a widely used, validated tool designed to assess sensitivity and evaluate cognitive status (Bassuk and Murphy, 2003). The 3MS examines functional domains, including orientation to space, person, and time, as well as short-term and long-term memory, attention, language processing, and visuospatial skills. A cut-off score of <80 out of 100 was used to define cognitive impairment, in line with prior clinical research (Salis et al., 2023). Although the 3MS has not been formally standardized in a South Indian elderly cohort, it has been widely adopted in several Indian hospital- and community-based cognitive screening studies due to its language adaptability (Lin et al., 2013). Participants with low scores were referred for expert clinical evaluation and categorized into two groups: those with cognitive impairment and those with cognitive normality (Figure 1).

### 2.7 Study group classification

Participants were divided into two groups: (1) individuals with diabetes and (2) individuals without diabetes. This division was based on medical history and HbA1c levels. The classification followed the American Diabetes Association criteria: Non-diabetic (HbA1c < 5.7%), Prediabetic (HbA1c 5.7%–6.4%), diabetic (HbA1c 6.5%–7.9%), and Uncontrolled diabetic (HbA1c ≥ 8.0%). This threshold allowed us to demarcate participants with persistent hyperglycemia and insulin resistance from those with adequate control, aligning with previous epidemiological studies evaluating metabolic-cognitive associations. Cognitive function was further classified using 3MS scores into two categories: cognitively normal (3MS ≥ 80) and cognitively impaired (3MS < 80). This classification allowed for subgroup analysis to investigate the impact of glycemic status on cognitive performance and biomarker profiles.

### 2.8 Statistical analysis

Data were entered using Microsoft Excel 2019 and analyzed using JMP Pro Version 17 (SAS Institute Inc., USA). Descriptive statistics were used to summarize clinical and biochemical parameters. Normality of continuous variables were tested using the Kolmogorov–Smirnov test. Continuous variables were compared using one-way analysis of variance (ANOVA), and categorical variables were compared using the chi-square test. Spearman’s rank correlation was used for assessing non-parametric correlations between continuous variables. A p-value of less than 0.05 was considered statistically significant.

Binary logistic regression analysis was performed to identify predictors of cognitive impairment. The dependent variable was cognitive status (3MS < 80 = impaired; ≥80 = normal). Independent covariates included age, sex, fasting blood sugar, HbA1c, fasting insulin, HOMA-IR, total cholesterol, triglycerides, HDL, and VLDL levels. Variables demonstrating statistical significance in univariate analyses (p < 0.05) or biological plausibility were entered into a multivariable model using backward stepwise selection. Adjusted odds ratios (ORs), 95% confidence intervals (CIs), and model performance indices (AUC) were computed to assess the independent contribution of each covariate.

## 3 Results

### 3.1 Characteristics of study participants

This cross-sectional survey comprised a total of 396 individuals, including 165 males (41.66%) and 231 female participants (58.33%). The mean age of the study cohort was 63.45 years, with a range of 51–80 years. The population dispersion of the study group, categorized by age and sex, is presented in (Table 1). Within the study participants, 204 subjects (51.52%) were categorized as having diabetes based on their clinical history and biochemical parameters (HbA1c ≥ 6.5%). A comparative assessment of participant characteristics between the individuals with diabetes and individuals without diabetes is summarized in (Table 2). Statistically significant differences (p < 0.05) were found between these two groups in the following physiological and biochemical parameters: age, fasting blood sugar levels, fasting serum insulin levels, HOMA IR, HbA1c scores, total cholesterol, triglycerides, VLDL levels, and 3MS score. These observations show that individuals with diabetes had significantly altered metabolic and cognitive profiles compared to those of individuals without diabetes.

**TABLE 1:** Age and sex distribution among study participants.

Age groups (years)	Total subjects (n = 396)	Individuals without diabetes (n = 192, 48.49%)	Individuals with diabetes (n = 204, 51.52%)
51–60	n = 170/42.93% M = 69 F = 101	n = 63/37.05% M = 21 F = 42	n = 107/62.94% M = 50 F = 57
61–70	n = 166/41.92% M = 69 F = 97	n = 99/59.64% M = 48 F = 51	n = 67/40.36% M = 32 F = 35
71–80	n = 60/15.15% M = 27 F = 33	n = 30/50.0% M = 15 F = 15	n = 30/50.0% M = 12 F = 18

**TABLE 2:** Comparison of study participant characteristics between individuals with and without diabetes.

Parameters	Individuals with diabetes		Individuals without diabetes		“P” value
	Mean	SD	Mean	SD	
Age (years)	62.60	8.18	64.31	7.18	0.0037*
FBS (mg/dL) (70–100 mg/dL)	122.66	55.62	82.85	17.98	<0.0001*
Fasting insulin (mU/L) (2-10 mU/L)	9.65	5.54	7.22	3.31	<0.0001*
HbA1c (%) (<5.6%)	7.45	1.78	5.65	0.35	<0.0001*
HOMA IR (<1.0)	2.94	2.20	1.53	0.84	<0.0001*
Total cholesterol (mg/dL) (<200 mg/dL)	181.75	37.29	170.24	32.46	0.0020*
HDL (mg/dL) (>40 mg/dL)	45.34	7.63	46.72	7.84	0.0893
LDL (mg/dL) (<100 mg/dL)	105.01	24.86	102.78	24.61	0.2884
VLDL (mg/dL) (<30 mg/dL)	30.27	13.02	23.63	8.80	<0.0001*
Triglycerides (mg/dL) (<150 mg/dL)	156.84	59.37	124.65	39.73	<0.0001*

FBS, fasting blood sugar; HbA1c, Glycated hemoglobin; HOMA IR, homeostatic model of assessment of insulin resistance; HDL, High-density lipoprotein; LDL, Low-density lipoprotein; VLDL, Very low-density lipoprotein.

*p < 0.05 is considered statistically significant.

### 3.2 Prevalence of cognitive impairment in study population and its association with diabetes

Among 396 total participants, 159 (40.15%) exhibited cognitive impairment traits. Females accounted for a greater proportion of cases (n = 92; 57.86%) in comparison to males (n = 67; 42.14%), although this variance was not statistically significant at the 0.05 level. When stratified by diabetic status, cognitive impairment was markedly higher among individuals with diabetes who had poor glycemic control. Specifically, 108 individuals with diabetes (52.94%) had cognitive impairment, whereas 51 individuals without diabetes (26.56%) were cognitively impaired (Table 3).

**TABLE 3:** Cognition and gender distribution among the individuals with and without diabetes groups.

Study groups	Subgroups	Male	Female
Individuals without diabetes (n = 192, 48.48%)	Normal cognition (n = 141, 73.44%)	n = 62, 43.97%	n = 79, 56.03%
Male = 84 (43.75%) Female = 108 (56.25%)
	Impaired cognition (n = 51, 26.56%)	n = 22, 43.14%	n = 29, 56.86%
Individuals with diabetes (n = 204, 51.52%)	Normal cognition (n = 96, 47.06%)	n = 49 51.04%	n = 47, 48.96%
Male = 94 (46.08%) Female = 110 (53.92%)
	Impaired cognition (n = 108, 52.94%)	n = 45, 41.67%	n = 63, 58.33%

The Chi-Square Test of Independence demonstrated a significant association between diabetes and cognitive impairment (p < 0.05). The odds ratio of cognitive impairment was nearly 2.42 times greater in individuals with diabetes compared to that of individuals without diabetes (OR≈2.42). Even the relative risk of cognitive impairment was elevated, with individuals with diabetes being 1.67 times more likely to experience cognitive impairment than individuals without diabetes (RR ≈ 1.67).

### 3.3 Cognitive impairment pursuant to glycemic status

To assess the influence of diabetes in accordance to its severity, on cognitive functioning, participants were categorized into four glycemic subgroups based on their clinical history and HbA1c values: Individuals without diabetes (HbA1c<5.7%), Individuals with prediabetes (HbA1c 5.7%–6.4%), Individuals with diabetes (HbA1c 6.5%–7.9%) and Individuals with uncontrolled diabetes (HbA1c ≥ 8.0%). Group distribution was as follows: Individuals without diabetes: n = 89 (22.47%), Individuals with prediabetes: n = 103 (26.01%), Individuals with diabetes: n = 149 (37.63%), and Individuals with uncontrolled diabetes: n = 55 (13.89%) (Table 4). Statistical comparison of demographic and clinical variables across these groups revealed significant differences (p < 0.05) in age, fasting blood glucose (FBS), fasting insulin, HOMA-IR, HbA1c, total cholesterol, triglycerides, HDL, VLDL, and 3MS scores (Table 5).

**TABLE 4:** Age-wise distribution of individuals across glycemic groups.

Age groups (years)	Total subjects (n = 396)	Individuals without diabetes (n = 89)	Individuals with prediabetes (n = 103)	Individuals with diabetes (n = 149)	Individuals with uncontrolled diabetes (n = 55)
51–60	n = 170/42.93%	n = 40/44.94%	n = 23/22.33%	n = 77/51.68%	n = 30/54.55%
61–70	n = 166/41.92%	n = 40/44.94%	n = 59/57.28%	n = 49/32.89%	n = 18/32.73%
71–80	n = 60/15.15%	n = 9/10.11%	n = 21/20.39%	n = 23/15.44%	n = 7/12.73%

**TABLE 5:** Comparison of study participant characteristics between individuals without diabetes, prediabetes, diabetes and uncontrolled diabetes.

Parameters	Individuals without diabetes (n = 89)		Individuals with prediabetes (n = 103)		Individuals with diabetes (n = 149)		Individuals with uncontrolled diabetes (n = 55)		“P” value
	Mean	SD	Mean	SD	Mean	SD	Mean	SD	
Age (years)	61.05	8.316	64.727	8.756	61.468	9.196	60.688	8.580	0.0045*
FBS (mg/dL) (70–100 mg/dL)	79.042	16.667	86.386	17.568	105.900	34.054	187.666	69.896	<0.0001*
Fasting insulin (2-10 mU/L)	6.576	3.285	7.912	3.341	9.127	4.674	12.084	7.460	<0.0001*
HbA1c (%) (<5.6%)	5.354	0.243	5.908	0.187	6.658	0.722	10.204	1.649	<0.0001*
HOMA IR (<1.0)	1.339	0.771	1.724	0.871	2.416	1.564	5.174	2.885	<0.0001*
3MS score	80.327	19.333	73.964	21.694	73.158	17.499	69.458	17.299	0.004*
Total cholesterol (mg/dL) (<200 mg/dL)	170.816	31.537	169.736	33.003	181	34.883	184.833	46.129	0.008*
HDL (mg/dL) (>40 mg/dL)	47.194	7.408	46.382	8.118	45.924	7.272	43.25	8.596	0.033*
LDL (mg/dL) (<100 mg/dL)	103.014	25.927	102.456	22.854	106.739	23.571	100.054	28.735	0.268
VLDL (mg/dL) (<30 mg/dL)	24.904	10.476	23.416	8.143	27.506	10.351	39.433	18.412	<0.0001*
Triglycerides (mg/dL) (<150 mg/dL)	121.796	38.309	127.082	39.915	146.789	50.610	190.375	72.932	<0.0001*

FBS: fasting blood sugar; HbA1c: Glycated hemoglobin; HOMA IR: homeostatic model of assessment of insulin resistance; HDL: High-density lipoprotein; LDL: Low-density lipoprotein; VLDL: Very low-density lipoprotein.

*p < 0.05 is considered statistically significant.

**FIGURE 2:**
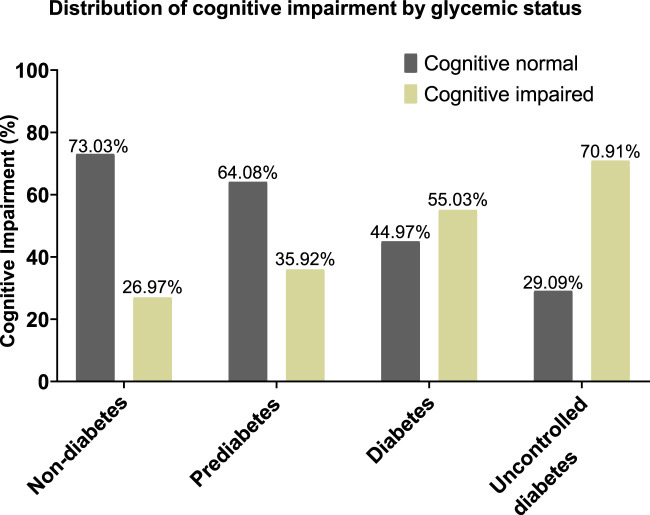
Bar graph representing the percentage of individuals in glycemic subgroups showing cognitive impairment.

The prevalence of cognitive impairment increased progressively with worsening glycemic status: Individuals without diabetes, 26.97% (n = 24 out of 89); Individuals with prediabetes, 35.92% (n = 37 out of 103); Individuals with diabetes, 55.03% (n = 82 out of 149); and Individuals with uncontrolled diabetes, 70.91% (n = 39 out of 55). In contrast, the ratio of subjects with normal cognition status declined drastically with rising glycemic dysregulation-from 73.03% in individuals without diabetes to just 29.09% in individuals with uncontrolled diabetes (Figure 2). This tendency was statistically significant (p < 0.05) as determined by the Chi-Square Test, indicating a stronger positive association between inconsistent glycemic status and cognitive impairment.

Abinary logistic regression analysis was conducted further to explore the association between glycemic status and cognitive impairment, using the individuals without diabetes as the reference. The results were as follows: Individuals with prediabetes had a nonsignificant increase in risk (OR≈1.56; p = 0.161). The individuals with diabetes had shown significantly higher odds of cognitive impairment (OR≈3.39; p < 0.001), and individuals with uncontrolled diabetes possessed the highest odds (OR ≈ 6.87; p < 0.001). These observations underscore the importance of glycemic control as a vital component in maintaining cognitive health among older adults. Among lipid profile variables, total cholesterol, triglycerides, and VLDL showed statistically significant increases with worsening glycemic status (p < 0.05), while HDL and LDL exhibited non-significant downward and marginal variations, respectively.

### 3.4 Cognitive impairment analysis by cognitive subdomains and its association with diabetes

Cognitive subdomain units such as delayed memory (p = 0.022), language ability (p = 0.002), and executive function (p = 0.007), have shown statistically significant differences among groups by Kruskal–Wallis’s test. Immediate memory and verbal skills did not show statistically significant differences across glycemic subgroups (p = 0.641 and p = 0.148, respectively), although a downward trend in mean scores was observed (Table 6).

**TABLE 6:** Comparison of cognition-subdomains between individuals without diabetes, prediabetes, diabetes and uncontrolled diabetes.

Parameters	Individuals without diabetes (n = 89)		Individuals with prediabetes (n = 103)		Individuals with diabetes (n = 149)		Individuals with uncontrolled diabetes (n = 55)		“P” value
	Mean	SD	Mean	SD	Mean	SD	Mean	SD	
Immediate memory	11.96	2.44	11.53	3.46	11.63	2.55	11.39	2.94	0.641
Delayed memory	9.55	2.79	8.45	2.93	8.76	2.75	8.26	2.76	0.022
Verbal skills	13.34	4.20	13.00	4.63	12.24	4.27	11.93	5.15	0.148
Language ability	15.49	3.94	15.15	3.67	14.15	3.67	13.09	3.84	0.002
Executive function	28.33	11.01	27.79	12.20	24.79	12.20	22.35	11.21	0.007

All assessed cognitive subdomains-namely, Immediate Memory (IM), Delayed Memory (DM), Verbal Skills (VS), Language Ability (LA), and Executive Function (EF)-were considered to be highly significant components of cognitive status. Among these components, Delayed memory has shown the strongest association with normal cognition, with an odds ratio of 211.54.

### 3.5 Gender-wise distribution of cognitive impairment according to glycemic subgroups

Gender-specific analysis revealed notable differences across glycemic subgroups: In the individuals without diabetes, more female participants (n = 16) had cognitive impairment than males (n = 8). In the Individuals with prediabetes, the dispersion was almost equal in all genders (females: 19; males: 18). Among individuals with diabetes, cognitive impairment was notably higher in females (n = 54) compared to males (n = 28). In Individuals with uncontrolled diabetes, this distribution was relatively equal (females: 20; males: 19). Conversely, among individuals with good cognition status, Females outnumbered males in both the individuals without diabetes and prediabetes. In the individuals with diabetes, males (n = 37) had normal cognitive scores compared to females (n = 30). Among individuals with uncontrolled diabetes, female predominance was seen, although the ratio is very minimal (females: 9; males: 7). Altogether, a higher prevalence of cognitive impairment was observed in females, particularly among individuals with diabetes (Table 7). However, this gender difference was not statistically significant (χ^2^ = 1.93, p = 0.165).

**TABLE 7:** Distribution of gender and cognitive impairment among individuals without diabetes, prediabetes, diabetes and uncontrolled diabetes.

Glycemic status	Cognitive impairment	Cognition normal
Individuals without diabetes	n = 24 (26.97%) Male = 08 Female = 16	n = 65 (73.03%) Male = 28 Female = 37
Individuals with prediabetes	n = 37 (35.92%) Male = 18 Female = 19	n = 66 (64.08%) Male = 30 Female = 36
Individuals with diabetes	n = 82 (55.03%) Male = 28 Female = 54	n = 67 (44.97%) Male = 37 Female = 30
Individuals with uncontrolled diabetes	n = 39 (70.91%) Male = 19 Female = 20	n = 16 (29.09%) Male = 07 Female = 09

### 3.6 Age-wise distribution of cognitive impairment

Age-specific analysis by Chi-square testing showed a significant association between cognitive impairment and age (χ^2^ = 30.49, p < 0.000001), with older individuals, notably those aged 71-80, showing a higher prevalence of cognitive decline. Logistic regression analysis revealed that participants aged 71–80 years were over five times more likely to exhibit cognitive impairment compared to those aged 51–60 years (p < 0.001). The 61–70 age group did not show a statistically significant difference (Table 8).

**TABLE 8:** Distribution of Age and cognitive impairment among individuals without diabetes, prediabetes, diabetes and uncontrolled diabetes.

Age groups	Cognitive impairment (n = 159)	Cognition- normal (n = 237)
51–60 years (n = 170)	n = 63/37.06%	n = 107/62.94%
61–70 years (n = 166)	n = 57/34.34%	n = 109/65.66%
71–80 years (n = 60)	n = 39/65%	n = 21/35%

### 3.7 Plasma amyloid biomarkers across glycemic categories

Table 9 explains the mean concentrations of Aβ 1-40, Aβ 1-42, and their ratio in four glycemic subgroups: non-diabetes, prediabetes, diabetes, and uncontrolled diabetes. Significant differences were observed in amyloid biomarkers across glycemic sub-categories. Aβ 1–40 levels were increased progressively with worsening glycemic control, reaching a peak in individuals with uncontrolled diabetes. Aβ 1–42 levels were markedly higher in individuals with diabetes and individuals with uncontrolled diabetes, compared to those of individuals without diabetes and prediabetes. The Aβ 1-42/Aβ 1-40 ratio was significantly elevated only in the diabetes group, but not in the uncontrolled diabetes group.

**TABLE 9:** Comparison of Serum amyloid levels between individuals without diabetes, prediabetes, diabetes and uncontrolled diabetes.

Parameters	Individuals without diabetes (n = 89)		Individuals with prediabetes		Individuals with diabetes		Individuals with uncontrolled diabetes (n = 55)		“P” value
	Mean	SD	Mean	SD	Mean	SD	Mean	SD	
Aβ 1-40 (pg/mL)	87.392	57.71	98.902	72.642	102.569	49.18	145.31	74.918	<0.0001*
Aβ 1-42 (pg/mL)	10.926	3.423	10.489	4.863	15.055	5.381	15.847	3.621	<0.0001*
Aβ 1-40/ Aβ 1-42 (pg/mL)	0.147	0.056	0.147	0.056	0.167	0.074	0.146	0.083	0.0001*

*p < 0.05 is considered statistically significant.

### 3.8 Correlation of 3MS score with clinical and biomarker parameters

Spearman’s rank correlation analysis was conducted to examine associations between Modified Mini- Mental State (3MSE) scores and various metabolic and amyloid-related biomarkers. The results are summarized in Table 10. Age and glycemic indices, as measured by FBS and HbA1c, were negatively correlated with 3MS scores, which were statistically significant. Fasting insulin showed no significant correlation, while HOMA-IR showed a weak but statistically significant negative association. Aβ 1–40 showed the strongest inverse correlation with cognitive performance. Aβ 1–42, although also negatively correlated, had a weaker association with the outcome. The Aβ 1–42/Aβ 1–40 ratio had a positive correlation with 3MS scores.

**TABLE 10:** Correlation between 3MS score and study parameters.

Parameters	Correlation co-efficient	“P” value
Age (years)	−0.2934	<0.0001*
FBS (mg/dL) (70–100 mg/dL)	−0.2272	<0.0001*
Fasting insulin (2-10 mU/L)	0.0232	0.6321
HbA1c (%) (<5.6%)	−0.4132	<0.0001*
HOMA IR (<1.0)	−0.1070	0.0270*
Aβ 1-40 (pg/mL)	−0.5102	<0.0001*
Aβ 1-42 (pg/mL)	−0.1725	0.0003*
Aβ 1-40/Aβ 1-42 (pg/mL)	0.2930	<0.0001*

The “r” values with statistical significance between the 3MS score and demographic and biochemical parameters.

*p < 0.05 is considered statistically significant.

### 3.9 Multivariable logistic regression predicting cognitive status

A multivariable logistic regression analysis was conducted to determine independent demographic and metabolic predictors of cognitive status, with normal cognition coded as one and cognitive impairment coded as 0. The model included age, sex, BMI, fasting glucose, fasting insulin, HOMA-IR, HbA1c, and lipid profile parameters (total cholesterol, HDL, LDL, triglycerides). The model demonstrated good discriminative performance (AUC = 0.81). The Nagelkerke R^2^ value of 0.343 indicated moderate explanatory power. The Hosmer–Lemeshow test showed statistical significance (χ^2^ = 17.30, p = 0.027), suggesting minor calibration deviation across predicted risk deciles.

After adjustment for all covariates, age, BMI, HbA1c, total cholesterol, and triglycerides were found to be independently associated with cognitive status. Age showed a strong inverse association with normal cognition (β = −0.060, OR = 0.94, 95% CI: 0.91–0.97, p < 0.001), indicating that each additional year of age reduced the odds of normal cognitive status by approximately 6%. BMI was positively associated with normal cognition (β = 0.093, OR = 1.10, 95% CI: 1.02–1.18, p = 0.015), suggesting that higher BMI modestly increased the likelihood of preserved cognition after adjustment. HbA1c demonstrated the strongest metabolic association (β = −0.579, OR = 0.56, 95% CI: 0.42–0.75, p < 0.001). Each 1% increase in HbA1c corresponded to a 44% reduction in the odds of normal cognition, indicating that chronic glycemic exposure is a major independent predictor of cognitive impairment. Among lipid parameters, total cholesterol showed a small but statistically significant positive association with normal cognition (β = 0.010, OR = 1.01, 95% CI: 1.00–1.02, p = 0.040), whereas triglycerides were inversely associated (β = −0.018, OR = 0.98, 95% CI: 0.98–0.99, p < 0.001), suggesting that higher triglyceride levels independently reduced the likelihood of normal cognition. Sex showed a borderline association (β = 0.484, OR = 1.62, p = 0.056), indicating a possible trend toward sex-related differences that did not reach statistical significance. In contrast, fasting glucose, fasting insulin, HOMA-IR, HDL, and LDL were not independently associated with cognitive status after multivariable adjustment (all p > 0.05) (Table 11).

**TABLE 11:** Adjusted logistic regression model predicting cognitive status.

Predictor	β value	Adjusted OR	95% CI	“P” value
Age (years)	−0.060	0.94	0.91–0.97	**<0.001**
Sex	0.484	1.62	0.99–2.66	0.056
BMI (kg/m^2^)	0.093	1.10	1.02–1.18	**0.015**
FBS (mg/dL) (70–100 mg/dL)	−0.003	1.00	0.98–1.01	0.712
Fasting insulin (mU/L) (2–10 mU/L)	−0.044	0.96	0.84–1.09	0.503
HOMA IR (<1.0)	0.338	1.40	0.87–2.25	0.162
HbA1c (%) (<5.6%)	−0.579	0.56	0.42–0.75	**<0.001**
Total cholesterol (mg/dL)	0.010	1.01	1.00–1.02	**0.040**
HDL (mg/dL)	0.013	1.01	0.98–1.05	0.443
LDL (mg/dL)	−0.009	0.99	0.98–1.00	0.203
Triglycerides (mg/dL)	−0.018	0.98	0.98–0.99	**<0.001**

HOMA IR, homeostatic model of assessment of insulin resistance; HbA1c, Glycated hemoglobin; HDL, High-density lipoprotein; LDL, Low-density lipoprotein; p < 0.05 is considered statistically significant (marked as bold).

Binary logistic regression analysis demonstrated a significant association between worsening glycemic status and reduced odds of normal cognition. Compared with non-diabetes individuals: Individuals with Prediabetes did not differ significantly in cognitive status (OR = 0.87, p = 0.663). Individuals with diabetes showed a 73% reduction in odds of normal cognition (OR = 0.27, 95% CI: 0.15–0.50, p < 0.001) and individuals with uncontrolled diabetes demonstrated an 88% reduction in odds of normal cognition (OR = 0.12, 95% CI: 0.05–0.27, p < 0.001). These findings indicate a graded deterioration in global cognitive performance with increasing severity of glycemic dysregulation. Multiple linear regression models adjusted for age, sex, BMI and lipid profile revealed domain-specific cognitive vulnerability.

Multiple linear regression models adjusted for age, sex, and BMI revealed domain-specific cognitive vulnerability. Immediate memory scores were not significantly associated with glycemic status. In contrast, delayed memory showed significant impairment in: Individuals with diabetes (β = −0.89, p = 0.009) and individuals with uncontrolled diabetes (β = −1.37, p = 0.002). Visuospatial performance demonstrated a progressive decline across glycemic categories, reaching statistical significance in uncontrolled diabetes (β = −1.56, p = 0.046) and approaching significance in diabetes (p = 0.059). Language scores were significantly reduced in: Diabetes (β = −1.26, p = 0.015) and Uncontrolled diabetes (β = −2.53, p < 0.001). Executive function demonstrated the largest effect size across all domains, with marked reductions in: Diabetes (β = −2.10, p = 0.035) and Uncontrolled diabetes (β = −5.66, p < 0.001).

## 4 Discussion

The present cross-sectional study evaluated the relationship between glycemic status, metabolic parameters, plasma amyloid biomarkers, and cognitive performance in older adults. The findings demonstrate a consistent and graded association between worsening glycemic status and increasing prevalence of cognitive impairment, accompanied by alterations in circulating amyloid-β biomarkers. Individuals with diabetes exhibited substantially higher cognitive impairment (52.94%) compared with individuals without diabetes (26.56%), and stratified analysis revealed a progressive rise across glycemic categories, reaching 70.91% among those with uncontrolled diabetes. This graded pattern aligns with epidemiological evidence showing that diabetes accelerates cognitive decline and increases dementia risk by approximately 1.5–2-fold (Biessels et al., 2006).

The multivariable logistic regression identified age, BMI, HbA1c, total cholesterol, and triglycerides as independent predictors of cognitive status (AUC = 0.81, Nagelkerke R^2^ = 0.343). These findings indicate that routinely measurable metabolic variables collectively explain a substantial proportion of cognitive variability, though residual variance likely reflects additional determinants such as genetics, inflammation, vascular health, and neurodegenerative pathology.

Age emerged as the strongest independent predictor, with each additional year reducing normal cognition odds by about 6% (Hou et al., 2019). This observation is consistent with extensive literature identifying aging as the principal determinant of neurodegenerative vulnerability due to cumulative oxidative stress, mitochondrial dysfunction, synaptic loss, and cerebrovascular injury (Mariani et al., 2005; Cicali et al., 2026). Notably, glycemic-group differences appeared less pronounced in the oldest participants, possibly reflecting age-related neuropathology overshadowing metabolic effects. Prior longitudinal studies have similarly shown that midlife metabolic dysfunction exerts stronger long-term cognitive consequences than late-life glycemic measures (Solomon et al., 2009). Sex showed only a borderline association after adjustment, indicating that metabolic and demographic factors may account for much of the observed sex variability. Nevertheless, hormonal, genetic, and sociocultural influences on cognition remain plausible and require further investigation. The predominance of female participants reflects global aging demographics from higher female life expectancy (Thorslund et al., 2013).

Among various metabolic factors, HbA1c showed the most significant link to cognitive function. A 1% rise in HbA1c was associated with a 44% decrease in the likelihood of maintaining normal cognitive abilities, highlighting that, prolonged hyperglycemic levels, rather than short-term metabolic changes, are the primary factors influencing cognitive decline. Chronic hyperglycemia promotes neuronal injury through multiple pathways including oxidative stress, advanced glycation end-products, mitochondrial dysfunction, endothelial injury, and neuroinflammation. Interestingly, fasting glucose, fasting insulin, and HOMA-IR were not independently associated after adjustment, suggesting that transient metabolic indices may have limited predictive value compared with cumulative glycemic burden. Similar findings have been reported in large cohort studies demonstrating stronger cognitive associations with HbA1c than with fasting glucose measures (Zheng et al., 2018; Xiao et al., 2025). The gradual decrease in the likelihood of normal cognitive function across different glycemic categories further reinforces a dose-responsive connection between metabolic dysregulation and cognitive decline. Such stepwise deterioration strengthens the biological plausibility of a causal metabolic contribution rather than a coincidental association.

Lipid parameters also contributed to risk of cognitive decline. Triglycerides were inversely associated with cognition, whereas total cholesterol showed a modest positive association. Elevated triglycerides are known to promote endothelial dysfunction, systemic inflammation, and cerebral small-vessel disease, all of which contribute to neurodegeneration (Paolini Paoletti et al., 2021). Conversely, the small positive association with total cholesterol may reflect complex age-dependent relationships; in older adults, very low cholesterol has sometimes been associated with frailty or preclinical neurodegeneration rather than protection. Although HDL and LDL were not independently significant after adjustment, dyslipidemia is strongly linked with insulin resistance, vascular dysfunction, and neuroinflammation, supporting the concept that global metabolic imbalance rather than isolated lipid fractions may drive cognitive decline (Yameny, 2025).

Domain-level cognitive status analyses revealed selective impairment patterns. Delayed memory and executive function showed the greatest decline across glycemic strata, whereas immediate memory and verbal abilities were relatively preserved. Executive dysfunction demonstrated the largest effect size, particularly in uncontrolled diabetes. This domain-specific vulnerability is consistent with evidence that metabolic disorders preferentially affect fronto-subcortical and hippocampal circuits (Yameny, 2025). Neuroimaging studies have demonstrated reduced hippocampal volume and impaired delayed recall in individuals with type 2 diabetes, supporting a structural basis for memory deficits (Milne et al., 2018; Moran et al., 2013). Cerebral insulin resistance may impair synaptic plasticity and neuronal glucose utilization, thereby disrupting memory consolidation (Spinelli et al., 2019; Duarte, 2023).

A notable strength of this study is the inclusion of plasma amyloid biomarkers. Elevated Aβ_1_–_40_ and Aβ_1_–_42_ levels were observed in individuals with controlled and uncontrolled diabetes, with increased Aβ42/Aβ40 ratio among those with uncontrolled diabetes. Experimental evidence indicates that insulin resistance alters amyloid precursor protein processing and promotes amyloidogenic pathways, linking metabolic dysfunction with Alzheimer-type pathology (de la Monte, 2012; Arrieta-Cruz and Gutiérrez-Juárez, 2016). Although MRI and CSF biomarkers remain the gold standard for detecting neurodegenerative pathology, circulating biomarkers offer a practical alternative for large-scale population screening (Kang et al., 2023). Plasma amyloid measurements have shown encouraging correlations with brain amyloid burden and PET imaging findings in large cohort studies, supporting their potential clinical utility (Pemberton et al., 2022). Importantly, participants with major systemic comorbidities were excluded during screening according to predefined eligibility criteria. However, detailed stratification of minor or subclinical comorbid conditions was not systematically recorded, and therefore residual confounding cannot be completely excluded. Medication status-including insulin, oral hypoglycemic agents, and lipid-lowering therapy was not incorporated into regression models because complete treatment data were not available for all participants. These factors may influence both metabolic parameters and cognitive outcomes and should be considered in future studies.

## 5 Limitations

The cross-sectional design precludes causal inference, and residual confounding from unmeasured variables such as education, socioeconomic status, physical activity, postmenopausal metabolic changes in females, genetic risk factors (e.g., APOE-ε4), and subclinical depression cannot be excluded. Known contributors to cognitive impairment, including hypertension and other vascular conditions, were not comprehensively assessed and may influence outcomes.

Hypoglycemic episodes were not directly evaluated; recurrent hypoglycemia is known to cause neuronal injury and structural brain changes. Future studies incorporating continuous glucose monitoring and glycemic variability indices would provide more comprehensive metabolic characterization.

Medication effects were not included in multivariable models due to heterogeneous treatment regimens and insufficient subgroup sizes. Longitudinal treatment-stratified studies are needed to evaluate pharmacologic influences on cognition.

### 5.1 Strengths

Despite these limitations, this study provides important population-specific data from an understudied South Indian cohort. Regional differences in genetics, diet, lifestyle, and healthcare can substantially influence metabolic and cognitive outcomes. By integrating glycemic indices, cognitive assessments, and amyloid biomarkers within a unified analytical framework, this study offers novel insights into the metabolic–neurodegenerative interface in aging populations.

## 6 Conclusion

Worsening glycemic control correlates with increased cognitive impairment and altered plasma amyloid profiles in older adults. Chronic hyperglycemia, indicated by HbA1c, is a major independent factor in cognitive decline, alongside age and lipid abnormalities. While causality is not established, the correlation between cognition and hyperglycemic severity suggests a metabolic-neurodegenerative continuum. These findings highlight the need for metabolic health to preserve brain function and the importance of cognitive screening for individuals with diabetes.

### 6.1 Future directions

Community-based screening can aid in early detection and counseling for high-risk populations. Longitudinal studies incorporating neuroimaging, CSF biomarkers, genetic profiling, and detailed vascular assessment are required to clarify mechanisms, establish temporal relationships, and determine whether metabolic optimization can slow cognitive decline.

## Data Availability

The raw data supporting the conclusions of this article will be made available by the authors, without undue reservation.
